# Associations between sexual and reproductive health knowledge, attitude and practice of partners and the occurrence of unintended pregnancy

**DOI:** 10.3389/fpubh.2022.1042879

**Published:** 2023-01-04

**Authors:** Ruping Liu, Xiaotong Dong, Xiaoning Ji, Shihan Chen, Qingqing Yuan, Yi Tao, Yaping Zhu, Sufang Wu, Jingfen Zhu, Yongbin Yang

**Affiliations:** ^1^Department of Obstetrics and Gynecology, Shanghai General Hospital, Shanghai Jiao Tong University School of Medicine, Shanghai, China; ^2^School of Public Health, Shanghai Jiao Tong University, Shanghai, China

**Keywords:** unintended pregnancy, partner, contraception, knowledge, attitude, practice

## Abstract

**Background:**

Although global contraceptive coverage has increased significantly, high rates of unintended pregnancy remain the current global status quo. A comparative analysis of the differences and correlations of knowledge, attitude and practice (KAP) of sexual and reproductive health (SRH) of both partners will help guide public health work according to gender characteristics and needs, and reduce the occurrence of unintended pregnancy.

**Methods:**

A questionnaire survey of people with unintended pregnancies including women and their male partners (*n* = 1,275 pairs) who sought help from the Shanghai General Hospital Affiliated to Shanghai Jiao Tong University School of Medicine from October 2017 to October 2021. Data were collected on sexual and reproductive health knowledge, attitudes, and practices in both partners who had unintended pregnancies. Chi-square test and Logistic regression were used to analyze the relationship between the occurrence of unintended pregnancy and KAP and its influencing factors. Paired odds ratio and McNemar's test were used to estimate the difference and concordance of KAP between partners.

**Results:**

This study included 1,275 partners with a mean age of 30.0 years. The partner's overall level of KAP is good. Compared with women, men had better knowledge (χ^2^ = 3.93, *p* = 0.047) and more active contraceptive practices (χ^2^ = 19.44, *p* < 0.001). In the analysis of partner concordance, male contraceptive intention was found to be better than female [matched pairs odds ratio (OR_MP_) = 2.56, *p* < 0.001], and the concordance of positive contraceptive practice between partners increased with male education [adjusted odds ratio (aOR) = 1.556, 95% confidence interval (CI) = 1.185–2.044, *p* = 0.001]. In partner-paired regression analysis, compared with good contraceptive knowledge in both men and women in the partner, the risk of negative contraceptive practice was 1.7 times (aOR = 1.721, 95% CI = 1.234–2.400, *p* = 0.001) higher with good contraceptive knowledge in women but negative in men, while women with poor contraceptive knowledge but men with good knowledge are 1.3 times (aOR = 1.349, 95% CI = 1.000–1.819, *p* = 0.05) more likely to have negative contraceptive practices. In addition, compared with partners with positive contraceptive attitudes, women with positive attitudes but negative men and women with negative attitudes but positive men had 1.7 and 1.4 times the risk of negative contraceptive practices, respectively.

**Conclusion:**

The study found that unintended pregnancy occurs mainly in young people, and the younger age of first sexual intercourse, the low education background and the lack of discussion of contraception between partners are risk factors for not taking contraceptive measures. Men's better knowledge and contraceptive practices compared with female partners, and poor male contraceptive knowledge and attitudes may lead to a higher risk of negative contraceptive practices, the results suggest that male KAP plays an important role in promoting contraceptive use and reducing unintended pregnancy.

## Introduction

Unintended pregnancy, including unwanted and mistimed pregnancies, is one of the major public health challenges and a major reproductive health problem (1). It has now affected the health, economic and social life of women and their families, and can lead to adverse social consequences, such as school dropout, violence and suicide, placing enormous physical, psychological and economic burdens on individuals, families and societies. In recent years, despite the almost universal use of condoms and other contraceptive methods, the incidence of unintended pregnancies has been increasing year by year (2). Worldwide, ~120 million unintended pregnancies occur each year, accounting for 40% of all pregnancies (3–6).

Studies have shown that most of these unintended pregnancy groups choose induced abortion to terminate the pregnancy, which has become a global phenomenon (7). The ever-increasing number of induced abortions not only has adverse consequences for women's physical and mental health (8), but in severe cases can lead to an increased risk of infertility, maternal mortality and neonatal mortality (9–13). According to statistics, worldwide, about 5 million women are hospitalized due to complications of unsafe abortion every year, and 47,000 women die from unsafe abortion (14), especially in less developed countries. In addition, the latest reports say that the US Supreme Court has overturned the 1973 Roe v Wade decision, ending women's 50 years old constitutional right to abortion and imposed comprehensive restrictions on abortion (15, 16), which may increase the incidence of unsafe abortion in the future. In fact, a multinational study found that countries with liberal abortion laws were more likely to offer safe abortion than countries that restricted abortion (14), and a hypothetical nationwide abortion ban would have catastrophic effects on maternal health (17). At the same time, in the United States, people have been relying on safe and legal abortion to cope with unintended pregnancies (18). However, the aftermath of this abortion ban suggests that women living in states that prohibit abortion may face unsafe abortions or may travel to another state or country to evade laws restricting abortion. It is clear that restricting abortion does not reduce its incidence, but rather reduces its safety (19), putting people's health, safety and property at great risk. Therefore, scientific contraception and reducing the occurrence of unintended pregnancy are particularly important.

The most common causes of unintended pregnancy are contraceptive failure and never use of contraceptive methods (20). Among them, the lack of contraceptive knowledge and the unreasonable choice of contraceptive methods are closely related to the increased risk of unintended pregnancy (21). Although people have a preliminary understanding of the existing contraceptive methods, the in-depth understanding of these methods is limited, or the misunderstanding of contraceptive knowledge will lead to an increase in the proportion of ineffective contraception (22), which will lead to the occurrence of unintended pregnancy. In addition, never use of contraceptive methods is also one of the main reasons for unintended pregnancy, and it can be seen from the Demographic and Health Survey (DHS) that the proportion of never use of contraceptive methods is high in developing countries (23), which is mainly related to the partner's negative contraceptive attitude, and of course to the partner's limited awareness of contraceptive methods, less sex education, and limited choice of effective contraceptives (24, 25). In conclusion, the importance of partners' knowledge, attitude and practice (KAP) of sexual and reproductive health (SRH) in reducing unintended pregnancies is well-documented. Proper knowledge and a positive attitude can increase partners' awareness of contraceptive practices and maximize the rational use of contraceptive methods, thereby preventing unnecessary risks.

Previous articles highlighted some of the current research aspects. First, in China and many other developing countries, research on the causes of unintended pregnancy and reproductive health services and education has focused primarily on women, exploring and documenting women's KAP of SRH is associated with the occurrence of unintended pregnancy (21, 26–29). In recent years, there has been renewed focus on the role of men in preventing unintended pregnancies (30, 31), and male attitudes and practices have been found to play a key role in contraceptive practice (30–33). And the World Health Organization and the United Nations and other organizations (34) mentioned in the global policy initiative on reducing unintended pregnancy that in clinical practice, health promotion and sexual health education, men's participation in decisions related to the prevention and management of unintended pregnancy should be considered. The aim is to encourage men to participate in discussions about contraception and pregnancy decisions.

In fact, decisions about sex, contraceptive use, and termination of pregnancy are rarely made by a woman or a man alone (35, 36), and partners' attitudes and practices also influence each other. Therefore, women and men are equally responsible for causing unintended pregnancy. However, there are few research data combined with both partners to analyze the causes of unintended pregnancy and explore the relationship between KAP of SRH and unintended pregnancy. It is known that in many countries, especially in China, women rely mainly on contraception and reproductive health decision-making and education are often also targeted at women (21). Although the use of contraceptive methods has increased significantly, the incidence of unintended pregnancy has increased year by year (37). Based on such a status quo, it shows that the focus of the past is only on women and not the key to solving the problem, and the previous research results that only used men or women as research objects were relatively one-sided. Therefore, this study conducted an in-depth comparison and analysis of the status of unintended pregnancy by both partners, aiming to jointly analyze the relationship between KAP of SRH and unintended pregnancy from the perspectives of men and women, and put forward gender-specific recommendations to reduce the number of unintended pregnancies, and improve women's reproductive health.

In China, with the release of second and third births, many people feel that “family planning” is outdated. However, according to the World Health Organization (WHO), family planning means “having the ability” to have the desired number of children at the interval and timing of births desired by individuals and partners through the use of contraceptive methods. All in all, the focus of family planning research both now and in the past is to ensure women's access to safe and effective comprehensive contraceptive measures, to protect women's reproductive health and fertility, and to strengthen high-quality fertility. The current status quo suggests that a key direction for the future is estimating the impact of the Coronavirus disease 2019 (COVID-19) pandemic on family planning indicators. Reports and early modeling efforts from administrative agencies and health systems suggest that large-scale disruptions to family planning services occurred in 2020 (38–41), affecting many people's need for abortion and contraception, and the long-term persistence of such changes is likely to result in change long-term fertility desires (42). There are reports that 60% of people's fear of COVID-19 is mainly hindered by the need for abortion, and 42% report that women are much less likely to seek an abortion during the pandemic, seriously affecting people's life plans (43). In addition, a qualitative study of young women under the age of 30 in six countries reported that among women who do not have access to abortion services due to COVID-19, some women choose to use medical abortions at home (44), and others seek unsafe abortions in local informal abortion facilities, increasing the incidence of complications such as infertility, reproductive system infections, and severe gynecological diseases, which can lead to maternal death. As a result, the overall level of contraceptive use reported in population-based surveys has increased to some extent as the pandemic has progressed, along with an increase in the demand for contraceptive and abortion information and a greater desire for access to knowledge (45, 46).

In addition, according to the World Health Organization and related research reports, during the new crown pneumonia pandemic, the provision of sexual and reproductive health guidance services in relevant medical departments was significantly reduced and interrupted (of different length and scale) (41, 47–50), but the proportion of the population in demand for reproductive health services increased from 23% in April 2020 to 40% in September 2020 (48). Therefore, exploring the relationship between the partner's KAP and unintended pregnancy, popularizing sexual and reproductive health knowledge and enhancing contraceptive determination have guiding significance for reducing the incidence of unintended pregnancy in the future.

## Materials and methods

### Study design and sample

This study was a retrospective questionnaire-based study of women and their male partners who sought help in the Department of Obstetrics and Gynecology, Shanghai General Hospital Affiliated to Shanghai Jiao Tong University School of Medicine for an unintended pregnancy. First, the investigator explained the purpose of the research study to the respondents and obtained informed consent. The Nurses explained precautions to women and their male partners and ensured the confidentiality of their participation in this study. The survey was conducted using an online self-administered questionnaire, including closed-ended and open-ended questions, and took 5–8 min. All questionnaires were conducted from October 2017 to 2021. The studies involving human participants were reviewed and approved by ethics committee of Shanghai Jiao Tong University School of Medicine (SJUPN-201718).

**Figure 1:**
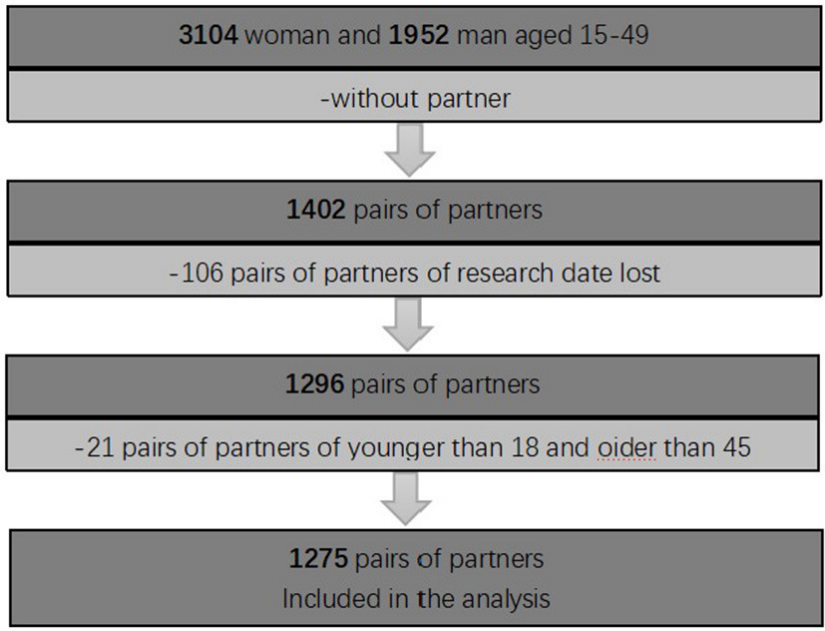
Description of the research sample.

A total of 3,104 females and 1,952 male partners aged 15–49 were collected in this study. The male and female partners were matched according to their names and basic information. Excluding males and females who failed to match successfully, there were 1,402 couples, of which 106 couples lost some information. As well as considering the universality of the research results, the age standard of the research subjects was controlled at 18–45 years old, a total of 21 couples were excluded, and the complete answers of 1,275 couples were finally included in the analysis. In the end, 1,275 couples' complete answers were included in the analysis, as shown in Figure 1. We define a partner as “two opposite-sex partners or couples.”

Research surveys included participants' sociodemographic characteristics and sexual and reproductive health. A self-made questionnaire was designed after reviewing previous similar surveys. Subsequently, the readability and comprehension of the questionnaire were verified. The validity of the questionnaire was checked by five experts in obstetrics and gynecology, and the questionnaire was discussed and adjusted with experts in public health and statistics, and content verification was completed in the final data analysis. By calculating the Cronbach's alpha value, which is 0.721, this means that there is acceptable reliability and consistency between test items.

### Categories

The final version of the questionnaire that collected the data consisted of four parts. The first part consists of six basic questionnaires, including age, gender, registration, education, profession, and income. The second part is about four items related to reproductive health, including the number of abortions, whether to discuss contraceptive matters, the age of first sexual intercourse, and the number of sexual partners. The third part includes seven questions related to contraception and abortion knowledge and aims to assess women's and men's sexual and reproductive health knowledge. The fourth part further evaluated the partner's contraceptive attitude and practice intention by answering three contraceptive attitude-related questions and three contraceptive practice questions.

### Measures

To assess participants' knowledge of sexual and reproductive health, participants were asked a series of questions about reproductive health knowledge, attitudes and practices. Among them, there are five questions about contraceptive knowledge, including “fertile periods of the menstrual cycle,” “rhythm contraception is an effective method of contraception,” “occasional unprotected sex won't lead to pregnancy,” “best time for emergency contraception,” “emergency contraceptives can be used frequently instead of oral contraceptives,” there are two questions about abortion knowledge, including “the harm of painless abortion is less than that with pain” and “abortion is harmful to the body.” There are three questions about contraceptive attitude, including “actively take contraceptive measures in the future,” “pregnancy can be avoided by taking proper contraceptive measures,” “contraception is troublesome, but worth it.” There are three questions on contraceptive practice, including “use contraception during sex,” “I can stick to contraception when my partner refuses it,” “I can stick to contraception.”

All questions use optional variables, and according to whether the answers were correct or not, the overall correct rate of each question, the correct rate of women and men respectively, and the overall response of each pair of partners were counted. For example, a question is “rhythm contraception is an effective method of contraception” and if the answer is “false,” then the question is recorded as the correct answer and is coded as “1.” If the answer is “correct,” “unclear,” or “haven't heard of the safe period,” the answer is recorded as wrong and coded as “0.” The cumulative total scores for KAP of SRH were calculated separately for men, women, and partners. Finally, with the median as the boundary, respondents with a total score at or above the median were defined as “good,” and those with scores below the median were defined as “poor.”

In addition, in order to assess the prevalence of partner concordance in KAP of SRH, according to whether the answers to questions related to reproductive health knowledge, contraceptive attitudes, and contraceptive practices were correct or not, we cross-tabulated the number (and percentage) of concordant and discordant answers among partners in four categories: both men and women in a couple answered correctly (W+M+); both men and women answered incorrectly (W-M-); women answered correctly, but men were wrong (W+M-); women answered incorrectly, but men were right (W-M+). Partner concordance on KAP was defined as practice in which both partners answered correctly or incorrectly on a question. Frequency and matched-pair odds ratio analyses were used to estimate patterns of partner concordance for KAP of SRH. The McNemar's test was used to determine the differences in KAP of SRH between male and female partners. “Paired” data were used in the estimation of the odds ratios and McNemar's test.

Furthermore, in order to calculate the concordance values for partner KAP of SRH, we coded the pattern “W+M+” as 1 and 0 otherwise. Univariate and multivariate logistic regression analyses were then used to calculate the matched pairs odds ratio (OR_MP_), and to test the statistical significance of the association between partner concordance in reproductive health knowledge, contraceptive attitudes and partner consistency in contraceptive practice. In addition, stratified analysis using multivariate logistic regression, according to the ages, registration and education of the couples, were used to confirm that the associations between partner concordance in reproductive health knowledge, contraceptive attitudes and partner concordance in contraceptive practice were consistent among different strata.

Finally, we recorded the total score of the answers to all questions about contraception and abortion knowledge, contraceptive attitude, and contraceptive practice, respectively, greater than the median as good, less than the median as poor, divided into four categories: that is, both women and men in couples were good (W+M+); both men and women were worse (W-M-); women were good, but men were worse (W+M-); women were worse, but men were good (W-M+). Univariate and multivariate logistic regression analyses were then used to calculate the odds ratios (ORs) and 95% confidence intervals (CIs) and to test the statistical significance of the association between partners in KAP of SRH.

### Analyses

After the data were exported as SPSS files from the Internet, the data were checked and sorted, and statistical analysis was carried out using IBM SPSS25.0 statistical software. Categorical variables were described using relative indicators such as rate and percentage. Statistical inference was performed using χ^2^ analyses, describing the demographic characteristics of 1,275 partners, using a multivariate logistic regression model to examine the association between participants' basic characteristics and KAP variables, reporting unadjusted and adjusted odds ratios (ORs and aORs) and 95% confidence intervals. Frequency and OR_MP_ analyses were used to assess partner concordance in KAP of SRH problems. Finally, after adjusting the variables of age, registration and education level of the partner, multivariate logistic regression was used for analysis. All statistical analysis tests were two-sided hypothesis tests, and the *p*-value < 0.05 was considered to be statistically significant.

## Results

### Socio-demographic characteristics

Table 1 shows the sociodemographic characteristics of the participants. A total of 1,275 pairs of partners (2,550 participants) were included and analyzed in this study. Among them, the average age of women is 28 years old, and the average age of men is 31 years old. Unintended pregnancy was most common among women (34.3%) between the ages of 18 and 25. The overall age of unintended pregnancy in men was higher than that in women, especially the number of unintended pregnancies in men over 35 years old (27.2%) was higher than that in women (16.4%). In addition, the study found that more than 41% of the participants were registered in rural areas, and most of the partners had a college education background or above, and they all had their own jobs and good incomes. Among them, it can be seen from the survey results that men's work income is significantly higher than that of women, with a significant difference (*p* < 0.001).

**Table 1:** Sociodemographic characteristics of the participants (*n* = 1,275 pairs).

**Variables**	**Total (*n* = 2,550)**	**Female (*n* = 1,275)**	**Male (*n* = 1,275)**	**χ^2^/*t***	***p*-value**
	***n*** **/%**	***n*** **/%**	***n*** **/%**		
**Age (years)**					
18–25	737/28.9	437/34.3	300/23.5	72.622	< 0.001
26–30	724/28.4	361/28.3	363/28.5		
31–35	533/20.9	268/21.0	265/20.8		
36–40	350/13.7	152/11.9	198/15.5		
41–45	206/8.1	57/4.5	149/11.7		
**Residence**					
Shanghai	793/31.1	357/28.0	436/34.2	11.569	0.003
Non-shanghai town	693/27.2	366/28.7	327/25.6		
Non-shanghai rural areal	1,064/41.7	552/43.3	512/40.2		
**Education**					
Middle school or less	379/14.9	206/16.2	173/13.6	4.038	0.133
High school	814/31.9	410/32.2	404/31.7		
College or higher	1,357/53.2	659/51.7	698/54.7		
**Profession**					
Works	1,744/68.4	834/65.4	910/71.4	35.991	< 0.001
Unemployed	196/7.7	138/10.8	58/4.5		
Other	610/23.9	303/23.8	307/24.1		
**Income**					
< 2,000	272/10.7	192/15.1	80/6.3	147.909	< 0.001
2,000–5,000	519/20.4	304/23.8	215/16.9		
5,000–8,000	821/32.2	447/35.1	374/29.3		
>8,000	938/36.8	332/26.0	606/47.5		

### Partner's response to KAP of SRH

Table 2 investigates issues related to contraception and abortion knowledge, contraceptive attitudes, and contraceptive practices. Overall, it can be seen that the overall practice of KAP by partners with unintended pregnancies is good. Among them, in terms of reproductive health knowledge, most partners believe that occasional unprotected sex won't lead to pregnancy (57.3% for women and 70.4% for men), and that Rhythm contraception is an effective method of contraception (67.4% for women and 54.5% for men), this wrong perception may be related to the weak knowledge of contraception and the psychology of luck. In addition, the correct rate of contraceptive knowledge about “fertile periods of the menstrual cycle” and “emergency contraceptive oral time” is significantly lower, indicating that people have a certain understanding of existing contraceptive methods, but their in-depth understanding of these methods is limited. As for the knowledge of abortion, although more than half of the partners believe that an abortion is harmful to the body (65.9% of women and 67.6% of men), a considerable number of people (80.6% of women and 78.3% of men) believed that painless abortion was less harmful to the body than painful abortions. This misperception of abortion methods may lead to partners not paying enough attention to the use of contraceptive methods, which may increase the probability of unintended pregnancies. Overall, compared with women's knowledge level, men had better understandings of sexual and reproductive health knowledge (χ^2^ = 3.93, *p* = 0.047).

**Table 2:** Partner responses to questions about KAP of SRH (*n* = 1,275 pairs).

**Practice variables**	Response^*^	**Total (*n* = 2,550)**	**Female (*n* = 1,275)**	**Male (*n* = 1,275)**	**χ^2^/*t***	***p*-value**
		***n*/%**	***n*/%**	***n*/%**		
**Knowledge of SRH**						
Occasional unprotected sex won't lead to pregnancy	Correct	922/36.2	544/42.7	378/29.6	46.813	< 0.001
	Wrong	1,628/63.8	731/57.3	897/70.4		
Rhythm contraception is an effective method of contraception	Correct	996/39.1	416/32.6	580/45.5	44.312	< 0.001
	Wrong	1,554/60.9	859/67.4	695/54.5		
Emergency contraceptives can be used frequently instead of oral contraceptives	Correct	1,844/72.3	836/65.6	1,008/79.1	57.947	< 0.001
	Wrong	706/27.7	439/34.4	267/20.9		
Fertile periods of the menstrual cycle	Correct	824/32.3	425/33.3	399/31.3	1.212	0.271
	Wrong	1,726/67.7	850/66.7	876/68.7		
Best time for emergency contraception	Correct	829/32.5	436/34.2	393/30.8	3.305	0.069
	Wrong	1,721/67.5	839/65.8	882/69.2		
The harm of painless abortion is less than that with pain	Correct	524/20.5	247/19.4	277/21.7	2.162	1.141
	Wrong	2,026/79.5	1,028/80.6	998/78.3		
Abortion is harmful to the body	Correct	1,702/66.7	840/65.9	862/67.6	0.855	0.355
	Wrong	848/33.3	435/34.1	413/32.4		
**Attitude of SRH**						
Actively take contraceptive measures in the future	Yes	2,296/90.0	1,188/93.2	1,108/86.9	27.984	< 0.001
	No	254/10.0	87/6.8	167/13.1		
Pregnancy can be avoided by taking proper contraceptive measures	Yes	1,748/68.5	859/67.4	889/69.7	1.637	0.201
	No	802/31.5	416/32.6	386/30.3		
Contraception is troublesome, but worth it	Yes	2,177/85.4	1,104/86.6	1,073/84.2	3.018	0.082
	No	373/14.6	171/13.4	202/15.8		
**Practice of SRH**						
Use contraception during sex	Yes	1,580/62.0	853/66.9	727/57.0	26.415	< 0.001
	No	970/38.0	422/33.1	548/43.0		
I can stick to contraception when my partner refuses it	Yes	1,428/56.0	544/42.7	884/69.3	183.983	< 0.001
	No	1,122/44.0	731/57.3	391/30.7		
I can stick to contraception	Yes	1,735/68.0	839/65.8	896/70.3	5.859	0.015
	No	815/32.0	436/34.2	379/29.7		
**Summary of selected practice variables (median)**						
**Knowledge**						
Poor practice		1,015/39.8	532/41.7	483/37.9	3.930	0.047
Good practice		1,535/60.2	743/58.3	792/62.1		
**Attitude**						
Poor practice		1,012/39.7	532/41.7	522/40.9	1.475	0.225
Good practice		1,538/60.3	784/61.5	753/59.1		
**Practice**						
Poor practice		923/36.2	515/40.4	408/32	19.441	< 0.001
Good practice		1,627/63.8	760/59.6	867/68		

^*^For all the answers to the questions, the correct answer is recorded as correct, otherwise it is recorded as wrong.

In terms of contraceptive attitudes, both males and females had positive contraceptive attitudes. Compared with males, females were more determined to contraceptives (χ^2^ = 27.984, *p* < 0.001). Compared with female contraceptive behavior intentions, males are more active, and this difference is statistically significant (χ^2^ = 19.44, *p* < 0.001).

### Associations between sociodemographic and reproductive health characteristics and KAP of SRH among partners

This study used logistic regression analysis to determine the association between sociodemographic and reproductive health characteristics and KAP of SRH, as shown in Table 3. From the results of the study, it can be seen that the educational level of the partner and whether or not to discuss contraceptive matters are closely related to KAP of SRH. Among them, compared with the participants with higher education level, the risk of reporting poor contraceptive knowledge is higher among those with high school or less, which was twice as high as those with college education and above (female, aOR = 2.389, *p* < 0.001 and male, aOR = 2.461, *p* < 0.001). Compared with men and women who did not discuss contraception and had only one sexual partner, men and women who discussed contraceptive matters frequently and had multiple sexual partners had good knowledge of contraception. In addition, the study found that women's knowledge level was also related to the number of abortions. Compared with women who had experienced more than one abortion, women who had never had an abortion were 1.3 times more likely to report poor contraceptive knowledge (aOR = 1.301, *p* < 0.030). Finally, an analysis of male participants found that older age (aOR = 0.723, *p* = 0.013) and younger age at first sexual intercourse (aOR = 1.283, *p* = 0.048) were also risk factors for poorer contraceptive knowledge.

**Table 3:** Logistic regression for the associations between sociodemographic characteristics, reproductive health characteristics and KAP among Partners.

**Variables**	**Female**						**Male**					
	**Knowledge**		**Attitude**		**Practice**		**Knowledge**		**Attitude**		**Practice**	
	**aOR^a^**	***p*-value**	**aOR^a^**	***p*-value**	**aOR^a^**	***p*-value**	**aOR^a^**	***p*-value**	**aOR^a^**	***p*-value**	**aOR^a^**	***p*-value**
**Marital status**												
Not married vs. married (ref)	0.883	0.337	0.868	0.333	1.049	0.740	0.948	0.715	1.110	0.455	1.166	0.221
**Age (years)**												
< 29 vs. ≥29 (ref)	0.927	0.617	1.486	0.002	1.225	0.083	0.723	0.013	0.922	0.509	0.816	0.162
**Residence**												
Rural vs. urban (ref)	1.252	0.076	1.330	0.029	0.982	0.889	1.300	0.057	1.093	0.486	0.962	0.782
**Education**												
High school or less vs. college or higher (ref)	2.389	< 0.001	1.645	< 0.001	1.606	< 0.001	2.461	< 0.001	1.676	< 0.001	2.076	< 0.001
**Profession**												
Unemployed vs. works (ref)	1.017	0.901	1.317	0.028	0.839	1.197	1.024	0.862	0.991	0.948	1.283	0.065
**Income**												
< 5,000 vs. ≥5,000 (ref)	1.420	0.005	1.133	0.356	1.188	1.160	1.513	0.004	1.368	0.024	1.213	0.197
**Number of abortions**												
1 vs. ≥2 (ref)	1.301	0.030	0.843	0.174	0.927	0.537	0.767	1.147	0.670	0.018	0.501	< 0.001
**Discuss contraceptive matters**												
No vs. yes (ref)	1.458	0.004	1.341	0.027	1.629	< 0.001	1.991	< 0.001	1.265	0.093	2.026	< 0.001
**Age at first sexual intercourse**												
< 21 vs. ≥21 (ref)	1.082	0.538	1.048	0.724	1.093	0.471	1.283	0.048	0.974	0.826	1.127	0.351
**Number of sexual partners**												
1 vs. ≥2 (ref)	1.317	0.030	1.298	0.040	1.064	0.628	1.346	0.023	1.175	0.196	0.981	0.889

aOR, adjusted odds ratio.

^a^Adjusting for age, marital status, income, profession, residence, and educational attainment.

In the analysis of contraceptive attitudes, the study found that young women with rural registration and less education were at higher risk of reporting poor contraceptive attitudes, while women who often discussed contraceptive matters with their partners and had multiple sexual partners had more positive contraceptive attitudes. In addition, the study also found that contraceptive attitudes and practices were more negative among men who caused women to have more than one abortion.

### Partner concordance for KAP of SRH

Table 4 describes the patterns of sexual and reproductive health knowledge, contraceptive attitudes, and practices among partners, comparing differences between male and female partners. Among them, the correct rate for the question of “whether emergency contraceptives can be used frequently instead of oral contraceptives” is highly consistent (55.1%). Male partners were more likely than female partners to believe that emergency contraceptives should not be used frequently instead of oral contraceptives (OR_MP_ = 2.277, *p* < 0.001). At the same time, there are also many partners (45.5%) who agree that an abortion has a great impact on the body, and the error rates of the questions about “female fertile period” and “best time for emergency contraception” are relatively high (47.5 and 48.6%). In addition, some partners (39.5%) mistakenly believe that rhythm contraception is an effective contraceptive method, and more than half (64.4%) of the partners agree that painless abortion is less harmful to women. Finally, through a comprehensive analysis of the answers to sexual and reproductive health knowledge, it again shows that the male partner's knowledge of contraception is better than that of the female partner (OR_MP_ = 2.26, *p* = 0.031).

**Table 4:** Partner concordance for KAP of SRH, and comparisons among different patterns (*n* = 1,275 pairs).

**Variables**	**W+M+**	**W-M+**	**W+M-**	**W-M-**	**OR_MP_**	**McNemar's test**
	***n*/%**	***n*/%**	***n*/%**	***n*/%**		***p*-value**
**Knowledge of SRH**						
Occasional unprotected sex won't lead to pregnancy	178/14.0	200/15.7	366/28.7	531/41.6	1.291	< 0.001
Fertile periods of the menstrual cycle	154/12.1	245/19.2	271/21.3	605/47.5	1.403	0.271
Rhythm contraception is an effective method of contraception	225/17.6	355/27.8	191/15.0	504/39.5	1.672	< 0.001
Emergency contraceptives can be used frequently instead of oral contraceptives	702/55.1	306/24.0	134/10.5	133/10.4	2.277	< 0.001
Best time for emergency contraception	174/13.6	219/17.2	262/20.5	620/48.6	1.880	0.055
The harm of painless abortion is less than that with pain	70/5.5	207/16.2	177/13.9	821/64.4	1.569	0.139
Abortion is harmful to the body	580/45.5	282/22.1	260/20.4	153/12	1.210	0.367
**Attitude of SRH**						
Actively take contraceptive measures in the future	1,035/81.2	73/5.7	153/12.0	14/1.1	1.297	< 0.001
Pregnancy can be avoided by taking proper contraceptive measures	630/49.4	259/20.3	229/18.0	157/12.3	1.668	0.189
Contraception is troublesome, but worth it	949/74.4	124/9.7	155/12.2	47/3.7	2.321	0.072
**Practice of SRH**						
Use contraception during sex	582/45.6	145/11.4	271/21.3	277/21.7	4.103	< 0.001
I can stick to contraception when my partner refuses it	430/33.7	454/35.6	114/8.9	277/21.7	2.301	< 0.001
I can stick to contraception	611/47.9	285/22.4	228/17.9	151/11.8	1.420	0.013
**Summary of selected practice variables** ^*^						
**Knowledge**	521/40.9	271/21.3	222/17.4	261/20.5	2.260	0.031
**Attitude**	501/39.3	252/19.8	283/22.2	239/18.7	1.679	0.195
**Practice**	580/45.5	287/22.5	180/14.1	228/17.9	2.560	< 0.001

OR_MP_, matched pairs odds ratio; W+M+, both men and women in a couple answered correctly; W-M-, both men and women answered incorrectly; W+M-, women answered correctly, but men were wrong; W-M+, women answered incorrectly, but men were right.

^*^The total score of the answers to questions related to KAP of SRH, greater than the median is regarded as good, and less than the median is regarded as poor, for example, the total score of women's knowledge in couples is greater than the median, which is good, but the total score of men's knowledge is less than the median, which is poor, and finally recorded as W+M-.

Regarding contraceptive attitudes, there was a high concordance (81.2%) between partners on “actively take contraceptive measures in the future,” and female partners had more positive contraceptive attitudes than male partners, with a statistically significant difference (OR_MP_ = 1.297, *p* < 0.001). Nearly half (45.5%) of the partners agreed on the use of contraceptive methods in future sex and were able to stick to contraception together. Among them, male partners had better contraceptive intention than female partners (OR_MP_ = 2.56, *p* < 0.001). On the whole, both partners have positive attitudes toward contraception, and the concordance is good, but the error rate concordance of some relatively professional common sense related to contraception is much higher than the accuracy rate concordance, which shows that partners' in-depth knowledge of sexual and reproductive health knowledge is weak, leading to an increased risk of contraceptive failure.

After adjusting for male and female age, registration, and education level, it can be seen from Table 5 that the concordance of active contraceptive practice between partners may increase with the education level of male partners (aOR = 1.556, 95% CI = 1.185–2.044, *p* = 0.001). It shows that the higher the male education level in the partner, the greater the partner's determination to use contraception together. Among them, from the analysis results of contraceptive attitudes, it can be seen that compared with partners with negative contraceptive attitudes, the probability of positive contraceptive practices increases by 2.9 times for couples who are more determined to contraception in the future (aOR = 2.953, 95% CI = 2.103–4.148, *p* < 0.001).

**Table 5:** Logistic regression of associations between knowledge, attitudes of SRH, and contraceptive practice among partners.

**Variables**	**Crude**			**Multivariate**		
	**OR**	**95% CI**	***p*-value**	**aOR^a^**	**95% CI**	***p*-value**
**Woman's age (years)**						
< 29 vs. ≥29 (ref)	1.267	1.014–1.583	0.037	1.215	0.969–1.522	0.092
**Man's age (years)**						
< 29 vs. ≥29 (ref)	1.054	0.845–1.315	0.642	0.811	0.600–1.097	0.174
**Woman's residence**						
Rural vs. urban (ref)	1.324	1.059–1.654	0.014	1.019	0.782–1.327	0.891
**Man's residence**						
Rural vs. urban (ref)	1.237	0.988–1.549	0.063	0.899	0.692–1.168	0.425
**Woman's education**						
High school or less vs. college or higher (ref)	1.799	1.413–2.290	< 0.001	1.232	0.930–1.631	0.146
**Man's education**						
High school or less vs. college or higher (ref)	1.868	1.461–2.388	< 0.001	1.556	1.185–2.044	0.001
**Summary of selected practice variables** ^*^						
**Contraceptive knowledge**						
Others vs. W+M+ (ref)	1.947	1.557–2.434	< 0.001	1.477	1.164–1.875	0.001
**Abortion knowledge**						
Others vs. W+M+ (ref)	2.160	1.726–2.704	< 0.001	1.831	1.450–2.313	< 0.001
**Contraceptive attitude**						
Others vs. W+M+ (ref)	1.975	1.572–2.480	< 0.001	1.609	1.267–2.042	< 0.001
**Summary of selected practice variables** ^**^						
**Contraceptive knowledge**						
W+M+	Reference					
W+M-	1.512	0.999–2.287	0.05	1.721	1.234–2.400	0.001
W-M+	1.977	1.340–2.916	0.001	1.349	1.000–1.819	0.05
W-M-	2.895	2.062–4.065	< 0.001	2.412	1.696–3.431	< 0.001
**Abortion knowledge**						
W+M+	Reference					
W+M-	2.580	1.612–4.127	< 0.001	1.460	1.080–1.973	0.014
W-M+	1.737	1.087–2.776	0.021	2.223	1.649–2.998	< 0.001
W-M-	3.954	2.580–6.060	< 0.001	3.598	2.331–5.555	< 0.001
**Contraceptive attitude**						
W+M+	Reference					
W+M-	1.534	1.066–2.207	0.021	1.736	1.287–2.342	< 0.001
W-M+	1.878	1.297–2.721	0.001	1.433	1.053–1.949	0.022
W-M-	2.858	2.061–3.963	< 0.001	2.466	1.763–3.448	< 0.001

OR, odds ratio; CI, confidence interval; aOR, adjusted odds ratio.

^*^The questions of partners' contraceptive knowledge, abortion knowledge and contraceptive attitude were divided into two parts according to the total score, including the partners who performed well (W+M+) and other situations (W+M-/W-M+/W-M-).

^**^According to the total score, it is divided into four parts, W+M+, both women and men in partners were good; W-M-, both men and women were worse; W+M-, women were good, but men were worse; W-M+, women were worse, but men were good.

^a^Adjusted for age, residence and education level for men and women in partners.

In addition, in the analysis of contraceptive and abortion knowledge and contraceptive attitude and contraceptive practice, it can be seen from Table 5 that the good concordance of contraceptive knowledge among partners increases the probability of positive contraceptive practice partner concordance by 1.477 times (aOR = 1.477, 95% CI = 1.164–1.875, *p* = 0.001). The concordance of correct abortion knowledge and positive contraceptive attitude among partners increased the odds of positive contraceptive practice concordance by 1.831 and 1.609 times (aOR = 1.831, 95% CI = 1.450–2.313, *p* < 0.001; aOR = 1.609, 95% CI =1.267–2.042, *p* < 0.001). It shows that good contraceptive and abortion knowledge and positive contraceptive attitude of both partners will promote the use of contraceptive methods, while poor contraceptive and abortion knowledge and negative contraceptive attitude are the risk factors for not taking contraceptive methods in sexual life between partners.

Analyzing the relationship with contraceptive practice in partner models with different knowledge and attitudes about sexual and reproductive health, it can be seen that compared with partners with good contraception and abortion knowledge and positive contraceptive attitudes, poor contraceptive knowledge (aOR = 2.412, 95% CI = 1.696–3.431, *p* < 0.001), poor abortion knowledge (aOR = 3.598, 95% CI = 2.331–5.555, *p* < 0.001), and negative contraceptive attitudes (aOR = 2.466, 95% CI = 1.763–3.448, *p* < 0.001) in both partners were risk factors for reporting partners' negative contraceptive practice.

In a more detailed comparative analysis of partners, it can be seen that in terms of contraceptive knowledge, compared with good contraceptive knowledge in both men and women in the partner (W+M+), the risk of negative contraceptive practice was 1.7 times (aOR = 1.721, 95% CI = 1.234–2.400, *p* = 0.001) higher in the partner with good contraceptive knowledge in women but negative in men (W+M-), while women with poor contraceptive knowledge but men with good knowledge (W-M+) are 1.3 times (aOR = 1.349, 95% CI =1.000–1.819, *p* = 0.05) more likely to have negative contraceptive practices. It also further indicated that, compared with female partners, poor contraceptive knowledge of male partners may lead to a higher risk of negative contraceptive practice, and thus a greater risk of unintended pregnancy. That is, men with good contraceptive knowledge can promote the use of contraceptive methods. In addition, compared with couples with positive contraceptive attitudes (W+M+), the risk of negative contraceptive practice for women with positive attitudes but negative for men (W+M-) and for women with negative attitudes but positive for men (W-M+) were 1.7 and 1.4 times. That is to say, partners with only men with good knowledge and attitudes had a 0.3–0.4 times lower risk of negative contraceptive practice compared with only women who performed well. Therefore, men's positive contraceptive attitudes have a greater impact on the use of contraceptive methods. The difference is that in terms of abortion knowledge, compared with men who have good abortion knowledge, women's good abortion knowledge has a greater positive impact on contraceptive practice.

## Discussion

This study analyzed the relationship between KAP of SRH and the occurrence of unintended pregnancy from the unique perspectives of both partners. Through investigation and analysis, we found that the vast majority of partners believed that good contraceptive knowledge (aOR = 1.477), good abortion knowledge (aOR = 1.831) and positive contraceptive attitude (aOR = 1.609) were the positive promoting factors of partners' contraceptive practice. Among them, compared with women's knowledge level (58.3%), men have better knowledge level (62.1%). This is inconsistent with previous research on American women (26), which showed that men have lower levels of knowledge about contraception than women. Compared with female contraceptive behavior intention, male performance was more positive. One possible explanation is that the vast majority of studies in China have found that men perceive actual or potential unintended pregnancy as a life event that will bring them moral and material dilemmas (34). Or with the development of society, men's sense of responsibility increases, and women's reproductive health may be considered, so they have strong contraceptive intentions, and the understanding of contraceptive knowledge will be more serious and active than women. In terms of contraceptive attitudes, both men and women are more positive in their determination to use contraception in the future, but compared with men, women are more determined to use contraception in the future. This may be because the occurrence of unintended pregnancy is closely related to women's reproductive health, so women's attitude toward contraception is more positive.

Most previous similar studies have investigated the association of KAP with male or female sociodemographic and reproductive health backgrounds on a country-by-country basis (22, 34, 51–54). In this study, we conducted a more detailed analysis of whether men and women in each partner had good knowledge and attitudes, and explored the impact and association of knowledge and attitudes on contraceptive practice between men and women in partners. The results of the study found that among partners, compared with women, men with poor contraceptive knowledge and negative contraceptive attitudes had a higher risk of negative contraceptive practice, and thus a greater risk of unintended pregnancy. That is to say, men with good contraceptive knowledge and positive contraceptive attitude can promote the use of contraceptive methods, indicating that men play an important role in promoting contraceptive practice and reducing the occurrence of unintended pregnancy, and this is similar to the findings (22, 34, 51, 54). Therefore, it is particularly important to reduce the occurrence of unintended pregnancies by changing the previous gender norms of “contraception depends on women.” However, according to the latest research report, male contraceptive initiative is generally not high in the world (55), so future research directions should highlight the theoretical focus of intervention behaviors to promote male contraceptive use, and call for reducing the occurrence of unintended pregnancy by helping men move from anticipation or consideration to action when adopting contraceptive practice.

In addition, the study also found that partners' education and income levels were strongly associated with KAP among participants' demographic characteristics. People with higher education and income have better knowledge of contraception and abortion, and have more positive contraceptive attitudes and practices. A survey shows that 74 million (86%) of the 86 million unintended pregnancies worldwide occur in economically underdeveloped countries (56). Compared with high-income countries, low-income countries have less personalized counseling on the choice of contraceptive measures, and more limited sources of effective and easily accessible reproductive health knowledge and modern contraceptive methods (3). For example, people with economic difficulties often choose the method of rhythm contraception, so they are more likely to have unintended pregnancy.

It is not difficult to understand that the level of education can determine a person's differences in cognition of the same things, which not only affects whether to use contraceptives, but also affects the choice of contraceptive methods and the decision-making of unintended pregnancy. Low education level limits the ability of individuals to receive contraceptive knowledge and abortion hazards, while the higher the education level, the higher the relative income level, and the more likely they are to independently acquire contraceptive-related knowledge through multiple channels. This is similar to the results of previous studies (57, 58), reporting that educational background is one of the most important factors for low contraception and abortion knowledge.

At the same time, after adjusting the variables of age, registration and education level, it was found that the concordance of active contraceptive practice between partners may increase with the education level of men (aOR = 1.556), that is to say, the higher the education level of the male partner, the greater the partner's determination to use contraception together. This can be explained by the fact that people from low educational backgrounds are more likely to see pregnancy as a positive life event, an opportunity to transform into a mature and responsible parent, and thus have relatively negative contraceptive attitudes and practices. For those with higher education, they believe that unintended pregnancy is an obstacle to becoming a mature and responsible person, which will affect their career planning (34), so contraceptive attitudes and practices are more positive. In response to this finding, we need to popularize sexual and reproductive health knowledge among targeted populations. For example, compared with people with high education level, it is particularly important to implement education on sexual and reproductive health knowledge for people with low education background and low income, and stablish relevant policy systems to enable young people to contact and understand relevant knowledge early.

According to the results of the investigation on the causes of unintended pregnancy (result not shown), 53.5% of the population did not use contraceptive methods, 30.8% of the population became pregnant due to contraceptive failure, and 15.7% had unknown reasons, indicating that the main cause of unintended pregnancy was not using contraceptive measures. This is similar to the results of previous studies. Globally, about 21% of married or cohabiting partner use contraceptive methods (57), indicating that the vast majority of partners do not use contraceptive methods during sex. In developing countries, ~50–60% of the population does not use contraception (23). In sub-Saharan Africa, South, Central, and Southeast Asia, up to 87% of unintended pregnancies occur in women who do not use contraception (59), which may be related to people's lower educational background, limited awareness of contraceptive methods, less sex education, and limited choice of effective contraceptives (20, 24).

The survey results show that although the overall knowledge level of the investigators in this study is relatively good, the correct rate of individual basic common-sense questions is low, such as the fertile periods of the menstrual cycle and the best time for oral contraceptives. At the same time, the concordance analysis found that the error rate concordance of some relatively detailed knowledge of contraception is much higher than the accuracy rate concordance and more than half (64.4%) of the partners in the KAP of SRH concordance analysis on the knowledge of abortion agreed that the painless abortion is less harmful to the body than the painless one. This misconception about contraceptive methods can affect the use of a partner's method of contraception. In recent years, although painless abortion has been accepted by increasing number of women because of its advantages such as less pain during an operation and faster recovery after an operation, it might even take abortion as a routine remedy for contraceptive failure. However, studies have shown that the rate of repeated abortion after painless abortion was higher than that after traditional artificial abortion (11), which increased the risk of anesthesia accidents (60) and also the incidence of complications such as uterine cavity adhesion, genital tract infection, and secondary infertility (8). The short-term and long-term complications affected women's physical and mental health.

In addition, due to people's inherent ideas or just listening to the descriptions of their friends around them, they will also have some wrong perceptions, which will increase the incidence of unintended pregnancy. For example, older women over 40 years of age believe that their reproductive function has deteriorated and they cannot conceive, thus reducing the use of contraceptives, which is likely to lead to unintended pregnancy. Many mothers during lactation believe that they will not be pregnant without menstruation, and they do not need to take contraceptive measures. But in fact, during lactation, even if they do not have menstruation, they may ovulate, and ovulation means there is a risk of unintended pregnancy. It also shows that people lack in-depth understanding of relevant knowledge and lack relatively professional guidance on accurate information and knowledge related to contraception, which has been mentioned in multiple studies (58, 61). Therefore, professionals should be encouraged to attach importance to popularizing knowledge, such as increasing online or offline popularization, promoting convenient and free contraceptive services provided by health care providers, and increasing the popularization of contraceptive methods among pharmacy workers to those in need of contraception.

The use of contraceptive methods has increased substantially worldwide since 1970 (55), but the incidence of unintended pregnancies has increased year by year. This also shows that most partners who have unintended pregnancy are willing to contraception, but do not use contraceptives or take incorrect contraceptive methods due to lack of contraceptive knowledge, and thus have a higher risk of unintended pregnancy. At the same time, the study found that unintended pregnancy was most common in the youngest age group (18–25 years old; 34.3% of women and 23.5% of men), and more than half of the respondents had their first sexual intercourse under the age of 21. In recent years, both the age of gestation and the age of first sexual intercourse tend to be younger (62–64), showing a downward trend (65–69), which is similar to the results of several studies (58, 70–73). Moreover, the study also found that men with smaller AFSI had a lower understanding of reproductive health knowledge (*p* < 0.05), and a younger AFSI is associated with a higher risk of unplanned pregnancy, which is consistent with other studies conducted in China (73). This makes sense given that younger women have lower knowledge of contraceptive methods and have higher frequency of intercourse and fertility. This result may be related to school education patterns and parental educational philosophies regarding sexual and reproductive health in China. Sexual issues are highly individual and secretive, family sex education should be the enlightenment education at the stage of sex education. However, influenced by traditional moral values, parents often avoid talking about sexual behavior with their children, let alone educate them about sex. On the other hand, the school curriculum in China rarely involves sex education or takes it seriously. Therefore, the educational concept of Chinese parents should be improved, and early sexual health education should be carried out for students in school to encourage them to take a more serious attitude.

## Limitations

Our analysis has some limitations. First, in China, because sex-related issues are still a very sensitive topic, and participants must recall the past, there may be reporting bias in responses to KAP-related questions, especially among unmarried adolescents. Second, in qualitative studies, there is no uniform definition of contraception or partner communication, and findings are limited in terms of generalizability and risk of bias. The population of this study is limited to paired partners, excluding most of the people who have not been successfully paired, as well as too young and too old partners, and the reported results are relatively limited. Finally, the respondents in this study were matched couples, including unmarried and married couples, and no differential analysis was conducted. There are differences in needs and perceptions of issues between these two groups.

## Conclusion

Although the global contraceptive coverage rate has increased significantly in recent years, and the contraceptive penetration rate in China is as high as 80%, the high incidence of unintended pregnancy is still the current global status quo. This study illustrates the lack of sexual and reproductive health education for partners in China through partner profiles. The key role of men in promoting contraceptive use is proposed, while providing evidence for the importance of male KAP in reducing unintended pregnancies between partners, filling the gap in the literature on joint research of both men and women with unintended pregnancy in developing countries and even around the world. Studies have shown that compared with women, men have more reproductive health knowledge, more positive contraceptive practice, and men's knowledge and attitudes are more positive factors in promoting contraception between partners. It shows that in the reproductive health education, the comprehensive education of partners should be given priority, and gender characteristics and needs should also be taken into consideration, not only taking into account the relative weakness of women's knowledge, but also paying attention to the role and role of male KAP in avoiding unintended pregnancy. It promotes male initiative and implements it into the contraceptive action between partners, so that men can be guaranteed in terms of contraceptive responsibility and attitude. In addition, it is also critical to identify targeted strategies when popularizing sexual and reproductive health knowledge. This study found that the younger age of the partner, low educational background, and lack of in-depth knowledge of relevant knowledge are risk factors for unintended pregnancy. Therefore, targeted education should be carried out for low-educated and young people. While emphasizing early sex education in schools and families, the government and health departments should also expand popular methods and promote the concept of “scientific contraception to reduce unintended pregnancy.”

## Data availability statement

The original contributions presented in the study are included in the article/supplementary material, further inquiries can be directed to the corresponding authors.

## Ethics statement

The studies involving human participants were reviewed and approved by Shanghai Jiao Tong University School of Medicine. The patients/participants provided their written informed consent to participate in this study. Written informed consent was obtained from the individual(s) for the publication of any potentially identifiable images or data included in this article.

## Author contributions

JZ, YY, RL, and XD contributed to conception and design of the study. YY organized the database. RL and XD performed the statistical analysis. RL wrote the first draft of the manuscript. XD wrote sections of the manuscript. XJ, SC, QY, and YT participated in data collection. JZ, YY, YZ, and SW have oversight and leadership responsibility for the planning and execution of research activities. YY and XJ provided financial support. All authors contributed to manuscript revision, read, and approved the submitted version.
